# Oligodendrocyte Plasticity with an Intact Cell Body In Vitro

**DOI:** 10.1371/journal.pone.0066124

**Published:** 2013-06-07

**Authors:** Manabu Makinodan, Aya Okuda-Yamamoto, Daisuke Ikawa, Michihiro Toritsuka, Tomohiko Takeda, Sohei Kimoto, Kouko Tatsumi, Hiroaki Okuda, Yu Nakamura, Akio Wanaka, Toshifumi Kishimoto

**Affiliations:** 1 Department of Psychiatry, Nara Medical University, Nara, Japan; 2 Department of Anatomy and Neuroscience, Nara Medical University, Nara, Japan; 3 Department of Psychiatry and Neurology, Kagawa University, Kagawa, Japan; Hospital Nacional de Parapléjicos – SESCAM, Spain

## Abstract

Demyelination is generally regarded as a consequence of oligodendrocytic cell death. Oligodendrocyte processes that form myelin sheaths may, however, degenerate and regenerate independently of the cell body, in which case cell death does not necessarily occur. We provide here the first evidence of retraction and regeneration of oligodendrocyte processes with no cell death in vitro, using time-lapse imaging. When processes were severed mechanically in vitro, the cells did not undergo cell death and the processes regenerated in 36 h. In a separate experiment, moderate *N*-methyl-D-aspartate (NMDA) stimuli caused process retraction without apparent cell death, and the processes regained their elaborate morphology after NMDA was removed from the culture medium. These results strongly suggest that demyelination and remyelination can take place without concomitant cell death, at least in vitro. Process regeneration may therefore become a target for future therapy of demyelinating disorders.

## Introduction

In the active lesions of demyelinating disorders such as multiple sclerosis, oligodendrocytes are thought to undergo cell death [1]–[3]. The cell death phenomenon generally results in a situation called demyelination, which means that axons lose their myelin sheaths. Remyelination by regenerating oligodendrocytes ensues to some extent. It is generally accepted that the regenerating oligodendrocytes derive from oligodendrocyte precursor cells (OPCs), which are widespread in the central nervous system [4], [5]. These cellular turnovers undoubtedly underlie the pathophysiology of demyelinating diseases [1], [6]–[8]. However, oligodendrocytes have elaborate processes to make myelin sheaths, and the dynamics of such processes should be analyzed separately from that of the cell soma. For example, demyelination and remyelination could simply reflect the processes being degenerated and regenerated, respectively, in which case whole cells would not have to turn over. In line with this speculation, differential distribution and function of glutamatergic receptors have been reported: AMPA (α-amino-3-hydroxy-5-methyl-4-isoxazole propionic acid)/kainite receptor subunits are mainly expressed in the soma, whereas NMDA (*N*-methyl-D-aspartate) receptor subunits are found predominantly in the processes of oligodendrocytes [9]. Stimuli that are specific to NMDA receptors injure the processes but not the soma of oligodendrocytes in vivo [10], suggesting that the processes and the soma behave independently. Moreover, non-lethal oxidative stress leads to a rapid and reversible shortening of oligodendrocyte processes which is prevented by antioxidants [11]. In order to directly observe oligodendrocyte morphology reported as above, we performed sequential investigation using time-lapse microscopic imaging as previous studies commonly employed to prove morphological dynamics of cells such as microglia [12], and currently demonstrate that mechanical injury to processes can be overcome without accompanying cell death. We also found that moderate stimulation of NMDA receptors led to a marked retraction of oligodendrocytic processes, which regenerated after removal of the NMDA agonist.

## Materials and Methods

### Cell culture and Treatment

Oligodendrocyte cultures were prepared by methods described previously [13]. Briefly, all fetuses were collected from a pregnant rat at embryonic day 16 (E16). Their brains (8–12 brains) were removed and the cerebral cortices were dissected from the whole brain, followed by removal of meninges and blood vessels. The cerebral cortices were mechanically dissociated through 140-µm pore size stainless steel mesh immersed in Eagle’s minimum essential medium (EMEM) supplemented with 10% fetal calf serum (FCS). The triturated cells were then sieved twice through 70-µm pore size nylon mesh, and the dispersed cells were seeded onto poly-L-lysine-coated 90-mm diameter culture dishes at a density of 10^7^ cells/dish. After 7 days’ culture, the cells were passaged with 0.05% trypsin in PBS and were cultured for 2 more days in 10% FCS/EMEM at a density of 3×10^6^ cells per non-coated Petri dish (2nd passage). On the 2nd day of culture, the medium was replaced with serum-free chemically defined Dulbecco’s modified Eagle’s medium (DMEM) supplemented with 10 µg/ml insulin, 0.5 µg/ml transferrin, 100 µg/ml bovine serum albumin, 60 ng/ml progesterone, 16 µg/ml putrescent, 40 ng/ml sodium selenite, 60 ng/ml N-acetyl-L-cysteine, 5 µM forskolin and 10 ng/ml platelet-derived growth factor-AA (PDGF, PeproTec), and the oligodendrocyte precursor cells (OPCs) were cultured for another 2 days. To differentiate the OPCs, the cells were continuously cultured with 30 ng/ml T3, 40 ng/ml T4, 10 ng/ml neurotrophic factor-3 and without PDGF for 3–5 days. Next, oligodendrocyte processes were mechanically severed using a disposable scalpel (No. 11, Feather). In some experiments, NMDA (Sigma) in Hank's balanced salt solution (HBSS) or the equivalent volume of HBSS was added to the medium, to a final concentration of 5 mM, for the retraction of oligodendrocyte processes. After the retraction of oligodendrocyte processes in both experimental treatments (mechanical injury and NMDA stimulation), 10 ng/ml PDGF was added to the culture medium to examine whether cell division could occur after oligodendrocyte processes had retracted. All experiments were performed using cells from multiple pregnant rats.

### Static and Time-Lapse imaging

Static imaging was performed using a DS-Fi1 camera (Nikon). To track morphological changes of oligodendrocytes, we performed time-lapse imaging using. Biostation system (Nikon). This method makes it possible to obtain an exact trace of single oligodendrocytes.

### Immunocytochemistry

For immunofluorescence of myelin basic protein (MBP), cells were incubated with anti-MBP antibody (rat, 1∶500, Chemicon) for 30 min, and then incubated with anti-rat Alexa488 (horse, 1∶2000, Invitrogen) followed by mounting with VECTASHIELD Mounting Medium including 4',6-diamino-2-phenylindole (DAPI) (50 ng/ml, Sigma). Fluorescence microscopy was performed using a Leica DM IRB microscope. Fluorescence images were merged using Adobe Photoshop software.

### Morphometric analysis

Oligodendrocyte diameter was measured by a modified method previously reported [11], [14].

### Statistics

Statistical analysis was performed with one-way ANOVA followed by Newman-Keuls test.

### Ethics statement

Every surgery was performed under isoflurane/oxygen anesthesia and all efforts were made to minimize pain. All experiments were approved by the Ethics Review Committee for Animal Experimentation of Nara Medical University.

## Results

**Figure 1:**
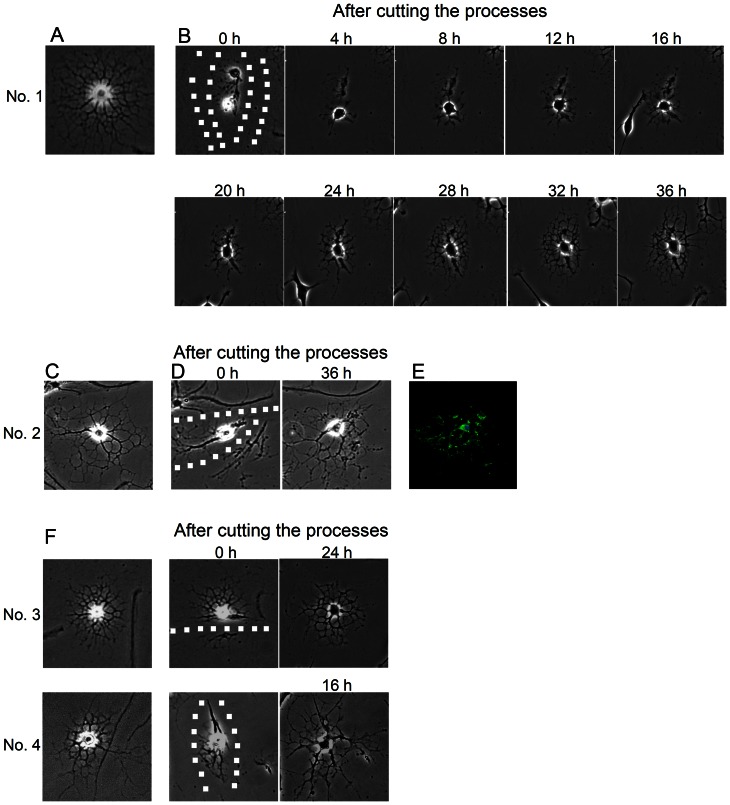
Processes of an oligodendrocyte gradually regenerate after severing. The processes of an untreated oligodendrocyte (A) were severed using a scalpel. After cutting, the processes gradually regenerated in 36 h (B). The processes of a second untreated oligodendrocyte (C) were also cut in the same way, and likewise regenerated in 36 h (D). The 36-h image of cell No. 2 viewed by immunofluorescence microscopy: MBP, green; DAPI, blue (E). Images of two additional oligodendrocytes (No. 3, No. 4) after their processes were severed are shown (F). The severed processes of No. 3 and No. 4 re-extended in 24 and 16 h, respectively. The severing tracks are dotted (B, D, F). ‘No.’ is an arbitrary reference number for individual oligodendrocytes.

We identified morphologically mature oligodendrocytes (Fig. 1A) in our mixed culture and confirmed that they expressed the mature oligodendrocyte marker MBP (data not shown). Using time-lapse microscopy, we monitored the morphological change of an oligodendrocyte after mechanically severing only the processes (Fig. 1B). The injured oligodendrocyte began to extend processes around 4 h after injury and regained an elaborate process morphology by 36 h after injury (Fig. 1B). We observed 10 mature oligodendrocytes, and none of them underwent cell death after injury in under our experimental conditions. The rate of process regeneration in response to severing varied from 16 to 36 h among individual oligodendrocytes (Fig. 1C–F), but all of them expressed MBP (Fig. 1E).

**Figure 2:**
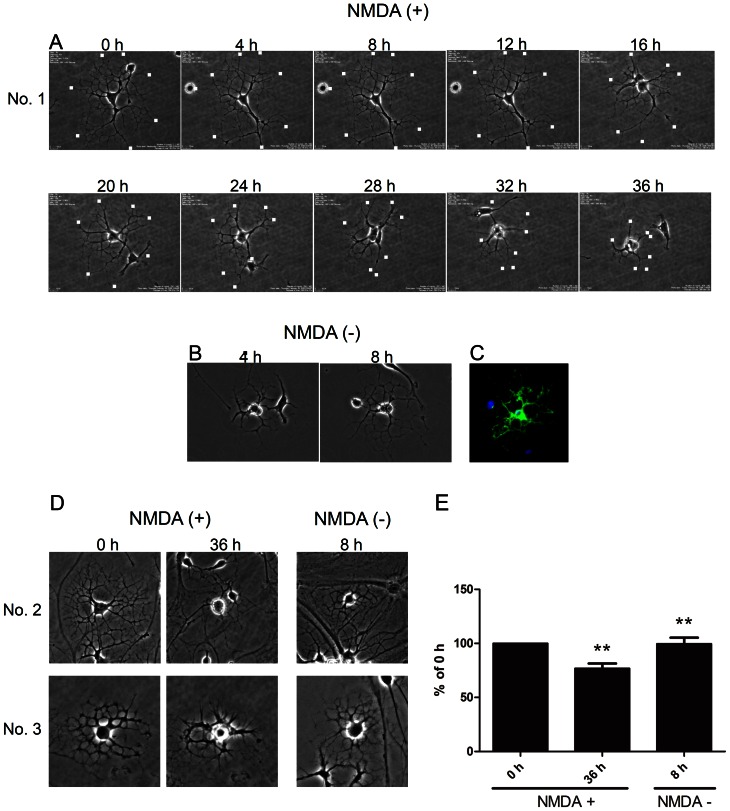
NMDA stimulation causes reversible retraction of oligodendrocyte processes. An untreated oligodendrocyte was stimulated with NMDA, which gradually caused retraction of the processes over 36 h, but the soma appeared intact. The leading edges of representative processes are marked by dots (A). The processes were progressively regenerated within 8 h after the removal of NMDA from the culture medium (B). The 8-h image of cell No. 1 viewed by immunofluorescence microscopy: MBP, green; DAPI, blue (C). Images of other representative cells (No. 2, No. 3), with NMDA and after its removal (D). Statistical analysis indicates that moderate NMDA treatment for 36 h significantly retract oligodendrocyte processes, which recovered 8 h after the removal of NMDA (E) (n = 5, each; ** p<0.01). ‘No.’ is an arbitrary reference number for individual oligodendrocytes.

Since the mechanical injury applied in the present study was not physiological, we next examined the effects of NMDA on the cultured oligodendrocytes. In various nervous system diseases, glutamate toxicity has been implicated in oligodendrocytic pathology as well as in neuronal death [15]–[18]. The exposure of cultured oligodendrocytes to NMDA for 36 h led to substantial retraction of processes, while the soma appeared intact (Fig. 2A, E). When we removed NMDA from the medium, the processes recovered their network structure in 8 h and expressed MBP (Fig. 2B, C, E). We traced seven oligodendrocytes under a time-lapse microscope; retraction and re-extension of processes with an intact soma were observed for five of them (Fig. 2D, two representative cases), while two died (data not shown).

## Discussion

Schwann cells, the counterparts of oligodendrocytes in the peripheral nervous system, are known to dedifferentiate and proliferate in response to injury [19]–[21]. We therefore anticipated that oligodendrocytes might also be able to divide after their processes were damaged. To test this possibility, in a separate time-lapse experiment, we re-administered PDGF, a potent mitogen for OPCs, in the culture medium immediately after the processes were severed. However, we observed no cell division within 36 h of injury (data not shown). Therefore, dedifferentiation of mature oligodendrocytes into OPCs appears unlikely, and the dynamics of oligodendrocytes and Schwann cells may thus be different. Taken together, these experiments indicate that oligodendrocytes have the potential to regenerate damaged processes without cellular turnover, at least in vitro.

Moderate NMDA stimulation can affect only the processes, and not the soma, of oligodendrocytes, and that its removal potentiates process regeneration. Since NMDA receptor subtypes are distributed predominantly in the processes, but not in the soma, and since NMDA receptors regulate Ca^2+^ concentration in the processes, but not in the soma [22], [23], it is possible that local Ca2+ concentration in a process is closely linked to its morphology. Furthermore, since NMDA receptors are expressed in central nervous system mitochondria [24] and mitochondrial inhibition alters oligodendrocyte morphology without cell death [11], mitochondrial dysfunction might be one of intriguing targets to pursue the mechanism of oligodendrocyte plasticity.

Recently, much attention has been paid to the plasticity of oligodendrocytes and myelination in adult brains without demyelination [25], [26]. Social experience-dependent plasticity of oligodendrocytes and myelination might be underlain by a mechanism without oligodendrocyte death since we did not detect any change of oligodendrocyte number in socially isolated mice [25] and social isolation increases oxidative stress [27], [28] along with an report that oxidative stress causes reversible retraction of oligodendrocyte processes with no cell death [11]. Although it remains to be tested whether the plasticity of oligodendrocyte processes takes place in vivo, mature oligodendrocytes may have greater potential to regenerate than has previously been thought. This notion may therefore provide an innovative concept for oligodendrocyte dynamics and a novel therapeutic approach in which remyelination could be achieved by stimulating process outgrowth from the intact somas of affected oligodendrocytes.
